# Chlorophyll fluorescence is a potential indicator to measure photochemical efficiency in early to late soybean maturity groups under changing day lengths and temperatures

**DOI:** 10.3389/fpls.2023.1228464

**Published:** 2023-10-23

**Authors:** Sonal Mathur, Beomseok Seo, Anjana Jajoo, Kambham Raja Reddy, Vangimalla R. Reddy

**Affiliations:** ^1^Adaptive Cropping Systems Laboratory, USDA-Agricultural Research Service (USDA-ARS), Beltsville Agricultural Research Center, Beltsville, MD, United States; ^2^School of Environmental and Forest Sciences, College of the Environment, University of Washington, Seattle, WA, United States; ^3^School of Biotechnology, Devi Ahilya University, Indore, India; ^4^Department of Plant and Soil Sciences, Mississippi State University, Starkville, MS, United States

**Keywords:** chlorophyll a fluorescence, day length (photoperiod), maturity group, photosystem II, soybean, temperature

## Abstract

In this study, we employed chlorophyll a fluorescence technique, to indicate plant health and status in response to changing day lengths (photoperiods) and temperatures in soybean early and late maturity groups. Chlorophyll a fluorescence study indicates changes in light reactions in photosystem II. Experiments were performed for 3-day lengths (12.5, 13.5, and 14.5 h) and five temperatures (22/14°C, 26/18°C, 30/22°C, 34/26°C, and 40/32°C), respectively. The I-P phase declined for changing day lengths. Active reaction centers decreased at long day length for maturity group III. We observed that low temperatures impacted the acceptor side of photosystem II and partially impacted electron transport toward the photosystem I end electron acceptor. Results emphasized that higher temperatures (40/32°C) triggered damage at the oxygen-evolving complex and decreased electron transport and photosynthesis. We studied specific leaf areas and aboveground mass. Aboveground parameters were consistent with the fluorescence study. Chlorophyll a fluorescence can be used as a potential technique for high-throughput phenotyping methods. The traits selected in the study proved to be possible indicators to provide information on the health status of various maturity groups under changing temperatures and day lengths. These traits can also be deciding criteria for breeding programs to develop inbreed soybean lines for stress tolerance and sensitivity based on latitudinal variations.

## Introduction

Soybean (*Glycine max* (L.) Merr) is a significant source of high-quality protein and oil for human consumption. It is the only dairy substitute and plant-based source for vegans. Soybeans are used extensively in the food industry as an animal feed and bioenergy resource. Over 80% of soybean production originates from the USA, Brazil, and Argentina (FAOSTAT, 2016). Abiotic factors such as temperature and day length affect soybean growth and development (FAS-USDA, 2020; Kimm et al., 2020; Kimm et al., 2021). With the continuous increase in temperature, the US soybean yield is projected to decrease by almost 20% before 2050 (Schlenker and Roberts, 2009; Kimm et al., 2021).

Chlorophyll (Chl), a fluorescence technique, has been used in plant physiology and has gained popularity in plant ecology, genetics, phenology, etc. (Maxwell and Johnson, 2000). Chl *a* fluorescence is the re-emission of light from plant chlorophyll molecules during photosynthesis. Thus, chlorophyll *a* fluorescence helps gather information about the changes in light-dependent reactions in plants (Frankenberg et al., 2011). It also provides insight into light absorption and conversion to biochemical energy (Kalaji et al., 2018). Chlorophyll *a* fluorescence is extensively used to understand photosynthetic energy flow and function under changing climate conditions such as high temperature (Mathur et al., 2011), drought, nitrogen deficiency (Congming and Jianhua, 2000; Huang et al., 2004), etc. Chlorophyll *a* fluorescence technique is nondestructive and noninvasive and helps to identify stress levels at any stage (vegetative and reproductive) of the plant (Maxwell and Johnson, 2000). Thus, without losing any leaf or plant organ, enormous information about plants can be obtained in a fraction of a second (measured between Fo and *F*_m_, Chl *a* fluorescence takes only a second per measurement), which makes it ideal for high-throughput phenotyping.

Photosynthesis is one of the primary targets for abiotic stress. Plant exposure to high temperatures (above 32°C–45°C, depending upon the region and country) leads to the destacking of grana, increases membrane fluidity, partially or fully the damaging oxygen-evolving complex (OEC), and modifies the ratio of ATP and NADPH (Herritt and Fritschi, 2020), ultimately impacting the dark reaction of photosynthesis by affecting RUBISCO activase, and carbon assimilation (Mathur et al., 2011; Snider et al., 2018). Previous studies using fluorescence measurements indicated that temperature stress caused increased heat dissipation and decreased the active reaction centers of the PSII. Soybeans are well known for their reduced yield due to high and low temperatures. Soybeans are considered short-day plants, meaning that their growth and reproductive processes, including flowering, pod development, and seed formation, are influenced by both the duration of daylight (photoperiod) and temperature. Growing soybeans successfully depends on specific factors like the amount of daylight they receive (photoperiod), temperature, and the right levels of rainfall, especially when they are germinating and flowering (Staniak et al., 2023). Exposure of soybean plants to low temperatures during the vegetative and reproductive stages leads to delayed flowering and prolonged vegetative stages. In contrast, exposure to high temperatures leads to decreased plant height, early flowering but aborted flowers and pods (due to temperature), and reduced photochemistry. High-temperature exposure during the seed filling stage results in reduced seed composition (Yang et al., 2023). Soybeans are also exposed to various weather conditions, such as cloudy days, rainy days, and fluctuations in daily temperatures. These conditions can decrease photosynthesis, increase the risk of infestations, prolong the vegetative stage, and potentially lead to delayed flowering and pod formation, among other effects (Silva et al., 2023). On the other hand, longer day lengths can lead to an increase in the number of leaves and nodes, potentially causing delayed flowering.

This study aimed to identify the difference in soybean maturity groups (early to late) based on chlorophyll *a* fluorescence measurement (lacking in the past years) and to make the traits indicators or deciding factors for tolerant and sensitive maturity groups. This comprehensive study details the light-dependent reactions of soybean early and late maturity groups in response to changing day lengths (photoperiods) and temperatures. The technique can help the crop plants detect early and late-stage stress levels without damaging them. The equipment is not confined to laboratories and can be used easily in fields, increasing its usability for farmers and breeders. This study will be helpful for climate change and improving soybean crop models. The study can be further developed for high-throughput phenotyping for a larger group of crops. We hypothesize that fluorescence measurements can bring new dimensions to precision agriculture for screening and developing stress-tolerant or sensitive soybean lines.

## Materials and methods

### Plant growth and cultivation

#### Plant material

Soybean (*Glycine max*) seeds were procured from the Germplasm Resources Information Network (GRIN, USA, https://www.ars-grin.gov/). Details about soybean maturity groups (MG) and procurement (accession number, etc.) can be obtained from **Table 1**. Experiments were conducted at the United States Department of Agriculture - Agricultural Research Service (USDA-ARS) facility in Beltsville, MD, USA. Plants were grown in the 4-gallon (0.015142 m^3^) pots. Three seeds were planted in each pot, later thinned to one plant per pot. Plants were regularly watered with a modified nutrient solution (×4 strength) (Yamakawa et al., 2004; Shimono and Bunce, 2009) to avoid water and nutrient stress.

**Table 1:** Name of soybean cultivars used in the study, maturity group, cultivar name, plant strain number, seed source, subcollection number, and origin.

Maturity group	Cultivar name	Strain	Seed source	Subcollection	Origin	Type
0	Early Sunrise	FC 32141	15U-5095-SD	Max	South Dakota, USA	Indeterminate
III	Ford	PI548562	09U-4322	Modern, 220121.1	Iowa, USA	Indeterminate
IX	Fusanari 1	PI416874B	14CR-1041-B	Max, 220109.1	Japan	Indeterminate
X	BRS Carnauba	PI675653	16CR-5	Max, 220109.2	Brazil	Determinate

The seeds were procured from the Germplasm Resources Information Network (ARS-GRIN, USA) (https://www.ars-grin.gov/).

### Plant growth chambers and treatment

The experiments were conducted in an indoor plant growth chamber (BioChambers, Bio Foot Series, Winnipeg, Manitoba, Canada) and an environmental growth chambers (EGC, Chagrin Falls, Ohio, USA) with varied day length and day/night temperatures.

### Day length (photoperiod) treatment

Plants were grown in EGC. Three-day lengths (photoperiods) were used, namely 12.5, 13.5, and 14.5 h. Relative humidity was adjusted to 70%. The plants were kept at a constant temperature of 26°C. The light intensity was controlled to be between 900 and 1,000 µmol m^−2^ s^−1^ (Bunce, 2016; Alsajri et al., 2019; Almeida et al., 2021; Alsajri et al., 2022; **Supplementary Figure S1**) at the top of the plant canopy during the experiment by adjusting a high-intensity discharge dimmer and bench height. Each chamber was dedicated to a 1-day length treatment. Plants were given the day-length (DL) treatment from planting/sowing until harvest. Pots were rotated regularly relative to each other to allow equal light intensities on all pots. Plants were supported with sticks for accurate growth and development.

### Temperature treatment

Plants were grown in BioChambers (Bio Foot Series; see above for details). Plants were grown at the set temperature from planting (sowing) until harvest. The chambers were set at day/night temperatures of 22/14°C, 26/18°C, 30/22°C, 34/26°C, and 40/32°C, respectively. The light intensity was controlled to be between 900 and 1,000 µmol m^−2^ s^−1^ (Bunce, 2016; Alsajri et al., 2019; Almeida et al., 2021; Alsajri et al., 2022) for all the chambers. Relative humidity (RH) was adjusted to 50% for chambers with high temperatures (34/26°C, 40/32°C), while RH was adjusted to 70% for chambers with temperatures of 22/14°C to 30/22°C, respectively. The day/night day length was set to 12.5/11.5 h for all the chambers. Each chamber was dedicated to one temperature. Pots were regularly rotated relative to each other to provide equal light intensities for all plants. Plants were supported with sticks for proper growth and development.

### Measurement of chlorophyll *a* fluorescence

Chlorophyll *a* fluorescence was measured using a Plant Efficiency Analyzer (Handy PEA) (Hansatech Norfolk, England, UK). Measurements were performed at the middle part (3 in. away from the tip and base of the leaf) of the fully opened and expanded top three to four leaves during different stages (vegetative and reproductive) of the plants. Plants were dark-adapted for a minimum of 40 min before the measurements. Excitation light of 650 nm (peak wavelength) from an array of three light-emitting diodes was focused on the leaf surface to provide homogenous illumination (for 1 s). The light intensity reaching the leaf was 3,000 μmol m^−2^s**^−^**^1^, sufficient to generate maximal fluorescence (*F*_m_) for all the treatments. The sensor head received the fluorescence signal during recording and digitized it in the control unit using a fast digital converter. Each plant, each maturity group, and each treatment underwent 15 measurements for each replicate. Leaves exhibited a polyphasic rise called O–J–I–P Chlorophyll *a* fluorescence transient; the minimum fluorescence O (50 µs PEA-based time mark) (Strasser et al., 1995) to J phase (ends at ~2 ms), the J to I phase (ends at ~30 ms), and the I to P phase (ends at ~500 ms). The O to J step represents a single turnover reduction due to the photochemical reduction of *Q*_A_− (Strasser et al., 1995; Stirbet, 2011; Kalaji et al., 2018; Tsimilli-Michael, 2020). J–I appears due to the reduction in the secondary quinone acceptor *Q*_B_, the PQ pool, and the cytochrome b_6_f complex (Lazar, 1999). At the end of point I, electrons reach plastocyanin and ferredoxin at the PSI’s electron acceptor side (Stirbet, 2011; Bussotti et al., 2020). The reduction continues as maximum fluorescence P is reached in less than 1 sec (Schreiber et al., 1994; Strasser and Strasser, 1995). **Table 2** provides details of various Chl *a* fluorescence parameters.

**Table 2:** Summary of various chlorophyll *a* fluorescence parameters, deexcitation parameters, energy fluxes, and cross-section (Schreiber et al., 1994; Strasser et al., 1995; Lazar, 1999; Chen and Cheng, 2009; Mathur and Jajoo, 2015; Snider et al., 2018; Stirbet et al., 2018).

Parameters	
OJIP	Inflection points at 20 or 50 µs, 2 ms, 20 ms, 500 ms
K	Additional band observed at 300–400 µs
*F*_o_	Minimal fluorescence
*F*_v_	Variable fluorescence
*F*_m_	Maximum fluorescence
*F*_v_/*F*_m_	Maximum quantum yield of primary photochemistry at *t* = 0
*F*_o_/*F*_m_ = (φ_Do_)	Quantum yield for heat dissipation of PSII
Deexcitation constants	
*K*_N_ = (ABS/CSo) × *K*_F_ × (1/*F*_m_)	Nonphotochemical rate constant
*K*_P_ = (ABS/CSo) × *K*_F_ × [(1/*F*_o_) − (1/*F*_m_)]	Photochemical rate constant
Yield, flux ratios (quantum efficiency)	
φ_Eo_ = ET_o_/ABS = ([1−(*F*_o_/*F*_m_) × (1−*V_j_*)	Quantum yield for electron transport (ET)
φ_Ro_ = RE_o_/RC = φ_Po_× ××δ_Ro_	Quantum yield of electron transport from *Q*_A_− to the PSI end electron acceptors
Ψ_E0_ = Et_o_/TR_o_ = 1−*Vj*	Probability (at time 0) that a trapped exciton moves an electron into the electron transport chain beyond *Q*_A_−
δ_Ro_ = RE_o_/ET_o_ = (1−*V_i_*)/(1−*V_j_*)	Efficiency with which an electron can move from the reduced intersystem electron acceptors to the PSI end electron acceptors
Density of RCs	
[γ(RC)/((1-γ(RC))]	Density of active reaction centers of PSII
Specific flux (per reaction center)	
TR_o_/RC = *M*_o_/*V_j_*	Maximum trapped exciton per RC at *t* = 0
ET_o_/RC = (*M*_0_/*V_j_*) × ψo = (*M*_0_/*V_j_*) × (1−*V_j_*)	Electron transport flux per RC beyond *Q*_A_− at *t* = 0
Performance/vitality Index and forces	
PI_ins_	Performance index (instrument, total), total PI, measuring up to PSI end electron acceptors
SF_abs_ = (Chl_Rc_/Chl_tot_) × φ _Po_ × ψo	Structural and functional index of plants
DF_total_	Total driving force for photosynthesis

### Plant total aboveground biomass measurement (dry mass)

Plant parts were separated into leaves, stems, and pods at harvest time. Plants were dried in a hot air oven at 70°C for 72 h until constant mass (Pierozan Junior et al., 2015) to measure the total above-ground biomass (dry weight). The total aboveground biomass was measured using an electronic balance (Sartorius Excellence, Germany) and (AND EK-12KA, Barford, MA, USA).

### Specific leaf area measurements

The specific leaf area (SLA) was calculated as described by Evans (1972) and Gunn et al (1999). It was measured as leaf area divided by leaf dry weight for the soybean maturity groups for each treatment.

### Data analysis and statistics

The data were analyzed using Origin ver.8.5 (Origin Lab Corporation, Northampton, MA, USA), GraphPad Prism 5.01 (GraphPad Software, Inc., La Jolla, CA, USA), and one-way analysis of variation (ANOVA) and the Duncan test were implemented by R package “agricolae” (de Mendiburu and Yaseen, 2020) in R, a language and environment (R Core Team, 2021). The significance was determined at ^***^p ≤ 0.001, ^**^p ≤ 0.01, and ^*^p ≤ 0.05. All the experiments were carried out in five replicates (five pots per treatment per maturity group). The heatmap was generated using the “ggcorrplot” package (version 0.1.4.) in R (version 4.2.2).

## Results and discussion

### Chlorophyll *a* transient curve for various maturity groups and day lengths

Photosynthesis is the only process that can convert solar energy into biochemical energy in the form of biomass. Chlorophyll *a* fluorescence studies were performed to quantify the photosynthetic behavior of early and late soybean maturity groups under various day lengths (photoperiods) and temperatures. Soybean leaves exhibited a polyphasic rise called the O–J–I–P fluorescence transient. We determined the effects of temperatures and day lengths on different maturity groups based on the transient shape. Each maturity group has a group-specific shape of fluorescence transient (OJIP), which is convenient for phenotyping that makes them specific to identify. Any alteration in the photosynthetic apparatus will change the shape of the transient for that maturity group.

**Figure 1:**
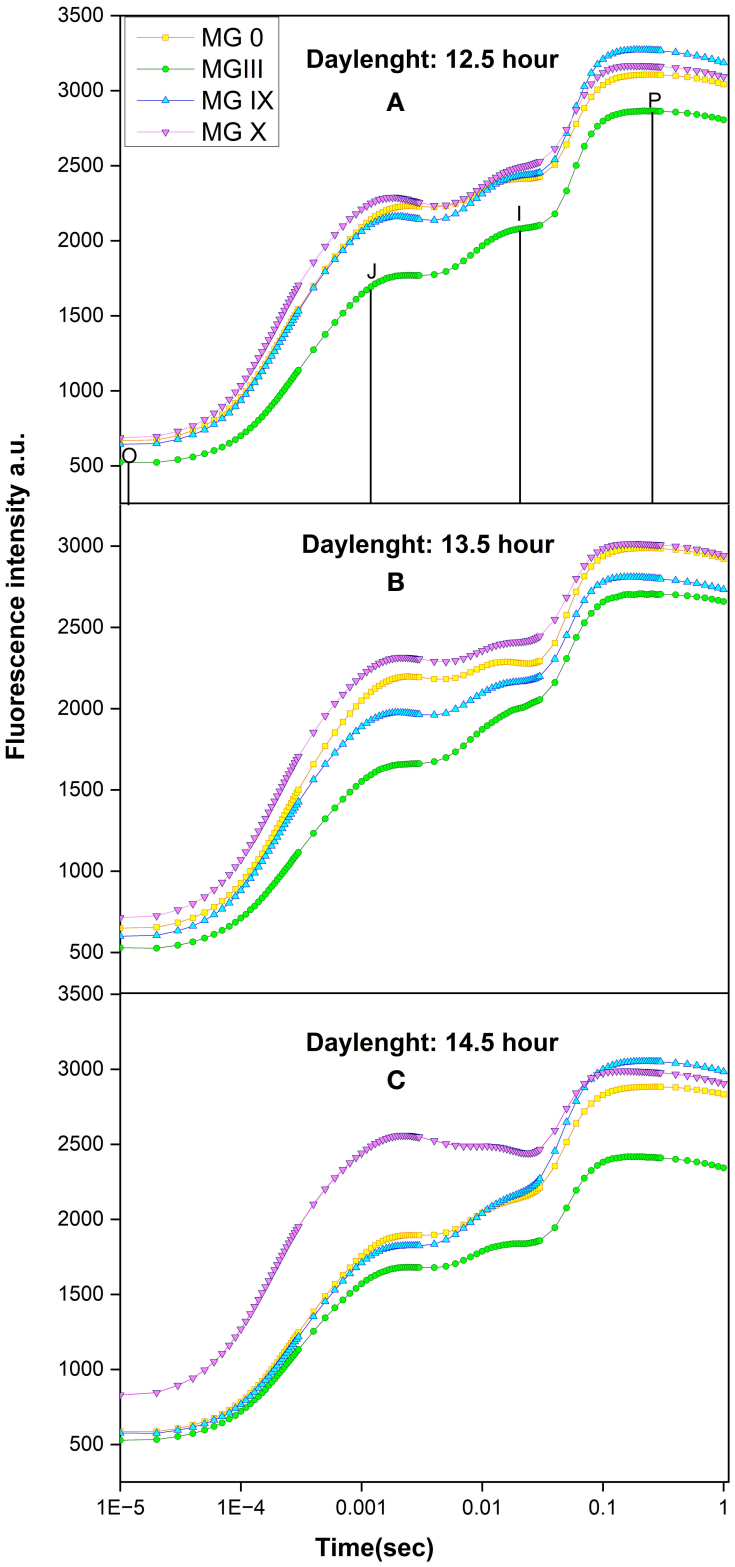
Chlorophyll *a* fluorescence transient for early to late soybean maturity group for **(A)** 12.5-h, **(B)** 13.5-h, and **(C)** 14.5-h day length.

**Figures 1A–C** present chlorophyll transients for different day lengths and maturity groups. Results suggested that a 13.5-h day length was critical for some maturity groups (0, III, X). Out of all the maturity groups, MG X showed increased minimal fluorescence (*F*_o_) and higher maximal fluorescence (*F*_m_) at 13.5- and 14.5-h DL (**Figures 1B, C**).

After 13.5-h day length, not many variations were obtained for some MGs. MG 0 and X are the most affected maturity groups at 13.5-h DL. A decline in maximal fluorescence was observed for MG 0, III. A decrease in the J–I phase was observed for MG 0, IX, and X at 13.5- and 14.5-h day lengths, indicating damage at the donor side of photosystem (PS) II. MG IX was the least affected maturity group at a 14.5-h day length. The transients’ shape for different maturity groups changed at 13.5- and 14.5-h DL. This indicates that even a small change in day length can affect plant photosynthesis, flowering, and soybean plant development, suggesting MGX to be more sensitive to any changes in environmental conditions. We observed that there were limited variations in many parameters once the day length exceeded 13.5 h. This indicates that the 13.5-h day length played an important role for the soybean maturity group X.

**Figure 2:**
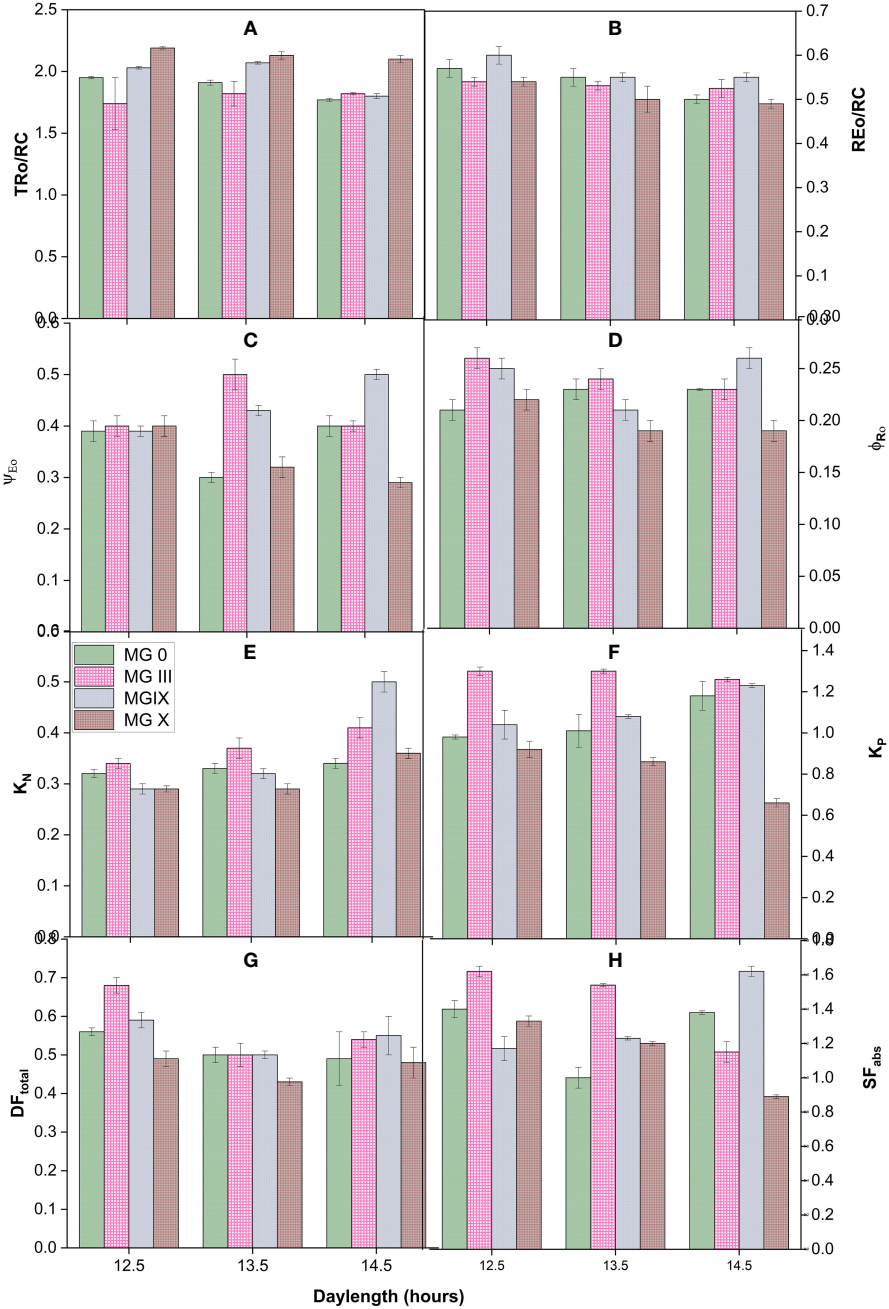
Measured flux ratios **(A, B)**, yield parameters **(C, D)**, deexcitation nonphotochemical and photochemical rate constants **(E, F)**, and vitality index and forces **(G, H)** for early to late soybean maturity groups under changing day lengths.

**Table 3** details how the light reactions are affected by different day lengths (photoperiods) in various maturity groups. The quantum efficiency of PSII is presented as *F*_v_/Fm. All the maturity groups depicted the highest efficiency at 12.5-h day length, while 13.5-h day length proved critical for the maturity groups (**Table 3**). Alteration for photosynthetic apparatus was observed at 13.5-h DL for MG 0 (evident by **Figure 1**). MG III showed significant changes for 13.5- and 14.5-h day lengths. Individually maturity group-wise, maximum variations for *F*_v_/*F*_m_ were observed for MG X at all day lengths. This change in *F*_v_/Fm indicated a decrease in the quantum efficiency of PSII under changing DLs. *F*_o_/*F*_m_ represents the maximum quantum yield for heat dissipation by PSII (ϕ_Do_) (Strasser et al., 1995). For MG 0, III, and X, *F*_o_/*F*_m_ increased at 13.5- and 14.5-h day length, suggesting that the plants were trying to regulate themselves with excess heat by dissipating extra energy in the form of heat, resulting in an increased *F*_o_/*F*_m_ (**Table 3**). φ_Eo_ decreased for MG 0 at a 13.5-h day length, indicating that the electron transport slightly reduced at the long day length (**Table 3**; **Figure 2**). This decrease in electron transport for other maturity groups also affects the yield. The less efficient the electron transport, the less the photoassimilate production and carbon allocation to various reproductive organs. MG X at longer day lengths showed a distinct decrease in linear electron transport, indicating less probability of the absorbed photons being transferred efficiently further to *Q*_A_−. MG IX was not affected much during the long day length. δ_Ro_ presents the efficacy or capability by which an electron can move from the reduced intersystem electron acceptors to the PSI end electron acceptors (Snider et al., 2018). δ_R0_ is dependent on electrons transferred from PQH_2_ to PSI. MG 0 and IX observed a less significant change, while MG III showed little more change for δ_Ro_. A considerable increase in MG X in δ_R0_ indicated that fewer or inefficient electrons were donated to reduce PQH_2_ at increased day lengths. This also suggests that PSI was not affected much for some maturity groups with changing day lengths. No significant change was observed for active reaction center density [γ(RC)/((1-γ(RC))] for MG III, but a significant change was observed for MG X at changing day lengths, indicating that DLs caused a downregulation in photon absorption, trapping, and ultimately affected the active reaction center of the PSII, which was also evident by a decrease in performance index (PI Inst.) (**Table 3**; **Figure 2C**). A decreased PI for MG X indicated decreased efficient absorption, transport, and utilization, which the energy pipeline model further supported. A significant change in all the fluorescence parameters was observed for MG X, which suggested that MG X was the most sensitive maturity group out of all the studied maturity groups studied, and this maturity group can be used as a reference maturity group for further breeding programs.

**Table 3:** Chlorophyll *a* fluorescence parameter for soybean maturity groups under various day lengths (photoperiods).

Maturity group	Day length (day/night) hour	*F*_v_/*F*_m_	*F*_o_/*F*_m_	ϕ_Eo_	δ_Ro_	PI Inst.	γ(RC)/((1-γ(RC))
0	12.5/11.5	0.813ab	0.19ab	0.28bc	0.84b	0.84c	0.42b
	13.5/10.5	0.800ab	0.24ab	0.23c	0.89a	0.77c	0.42b
	14.5/9.5	0.811a	0.18b	0.30a	0.68c	1.32a	0.47a
	Significance	^**^	^**^	^***^	^***^	^***^	^***^
	*p*-value	0.008	0.008	<0.001	<0.001	<0.001	<0.001
III	12.5/11.5	0.842a	0.16c	0.38a	0.70ab	1.92a	0.49
	13.5/10.5	0.829ab	0.17bc	0.39a	0.62b	1.79ab	0.47
	14.5/9.5	0.804c	0.20a	0.30b	0.77a	1.05bc	0.45
	Significance	^***^	^***^	^*^	^*^	^*^	ns
	*p*-value	<0.001	<0.001	0.027	0.028	0.048	0.384
IX	12.5/11.5	0.833a	0.17b	0.34b	0.74bc	1.19b	0.41b
	13.5/10.5	0.823ab	0.18ab	0.32bc	0.73cd	1.16b	0.41b
	14.5/9.5	0.835a	0.17b	0.40a	0.64d	1.86a	0.46a
	Significance	^**^	^**^	^***^	^***^	^***^	^**^
	*p*-value	0.005	0.005	<0.001	<0.001	<0.001	0.006
X	12.5/11.5	0.816ab	0.18cd	0.28a	0.73c	0.71ab	0.37
	13.5/10.5	0.792bc	0.21bc	0.23b	0.84bc	0.54bc	0.37
	14.5/9.5	0.748d	0.25a	0.25c	1.33a	0.23d	0.34
	Significance	^***^	^***^	^***^	^***^	^***^	^***^
	*p*-value	<0.001	<0.001	<0.001	<0.001	<0.001	<0.001

Significance was determined at ^***^p ≤ 0.001; **p ≤ 0.01; ^*^p ≤ 0.05. Mean with the same letter are not significantly different.

### Effect of day lengths on specific fluxes, yields, and efficiencies of different soybean maturity groups

Derived parameters, flux or yield ratios, specific fluxes, quantum yield and efficiencies, and deexcitation rate constants were studied for different soybean day lengths and maturity groups. **Figures 2A–H** present the yield and PSII efficiency traits (such as Ψ_E0_, φ_Ro_, REo/RC, and TRo/RC) (**Table 2**). Individually for the maturity groups, a gradual change was observed in trapping the reaction centers (TRo/RC). The trapping of reaction centers (TRo/RC) showed no significant difference for MG III, while a slight decrease was observed for MG 0 at 14.5 h. It decreased for MG IX at 14.5 h DL. The electron movement restricted the quantum efficiency. The apparent explanation for the decline may be that longer DLs increased the efficacy of reaction centers to reduce plastoquinone (Chen and Cheng, 2009; Snider et al., 2018). Day lengths of 13.5 and 14.5 h impacted the maturity groups resulting in a decreased ratio. MG IX and X depicted a slight decrease in the REo/RC ratio (**Figure 2B**). The electron movement restricted the quantum efficiency, suggesting that day lengths (photoperiods) did not cause much impact at the PSI end electron acceptor side. In support of **Table 3**, for MG 0 and X, 13.5-h day length was a critical day length that caused a significant impact, also observed from a decrease in Ψ_Eo_ and φ_Ro_ (**Figures 2C, D**). The values for Ψ_E0_ declined for MG 0 and X at 13.5-h day length, suggesting that the chance of an exciton to move an electron further than *Q*_A_− (Snider et al., 2018) decreased at this DL as compared to other day lengths. However, the traits did not decrease in MG III for a 13.5-h day length, which is supported by **Table 3** (φ_Eo_), indicating that electron transport was impacted but not till the PSI level. MG X showed the lowest values for electron transport further than *Q*_A_−. The minimum value for φ_Ro_ was also observed for MG X, suggesting that the quantum yield of electron transport from *Q*_A_− to PSI end electron acceptor decreased due to the longer DLs. Thus, these two parameters indicated that PSI was not affected much due to long day lengths for some of the maturity groups.

Deexcitation rate constants of the PSII antenna were studied by measuring *K*_P_ and *K*_N_ for all the DLs and maturity groups (**Figures 2E, F**). The deexcitation nonphotochemical rate constant (*K*_N_) increased for MG IX and X at longer DLs. In contrast, not much change was observed for other MGs. MG 0, IX showed an increase in photochemical rate constant (*K*_P_), while it decreased for MG X. However, there was little change for MG III (**Figure 2E**). This suggests that plants did not try to release excess energy in the form of heat at short DLs, and maximum photochemistry was taking place (**Figures 2E, F**).

Furthermore, the driving force for photosynthesis (DF_total_) (Srivastava et al., 1999; Mathur and Jajoo, 2015) and structural-functional index (SF_abs_) of plants (**Figures 2G, H**) (Dudeja and Chaudhary, 2005; Mathur and Jajoo, 2015) were studied for all the DLs and maturity groups. Both these parameters represent the health status and viability of the plants. To enumerate the potential of plant photosynthesis, DF_total_ was studied for all the DLs and maturity groups. All the maturity showed a decrease for DF_total_ at 13.5 and 14.5 h but individually, MG 0 showed a significant decline at the long day lengths. Each maturity group showed maximum efficiency at 12.5-h DL. These results suggested that DF_total_ can be considered one of the indices for studying responses for different maturity groups at various photoperiods. SF_abs_ is the combined presentation of absorbance, density of reaction centers, electron transport, and quantum efficiency of PSII. Results obtained from SF presented that absorption, the density of reaction centers of PSII, and electron transport were efficient for all the MGs. All the MGs showed the highest values of SF_abs_ at 12.5 h of day length. Individually, MG III and X showed a maximum decline for SF_abs_, while it was not stable for MG 0. The increase of MG IX at 14.5-h day length may be due to the absorption and accumulation of excess inefficient reaction centers and getting converted from active to lesser active PSII reaction centers (as observed from energy pipeline leaf models also) (**Figure 2H**).

### Impact of temperature treatment on early and late soybean maturity groups

**Figure 3:**
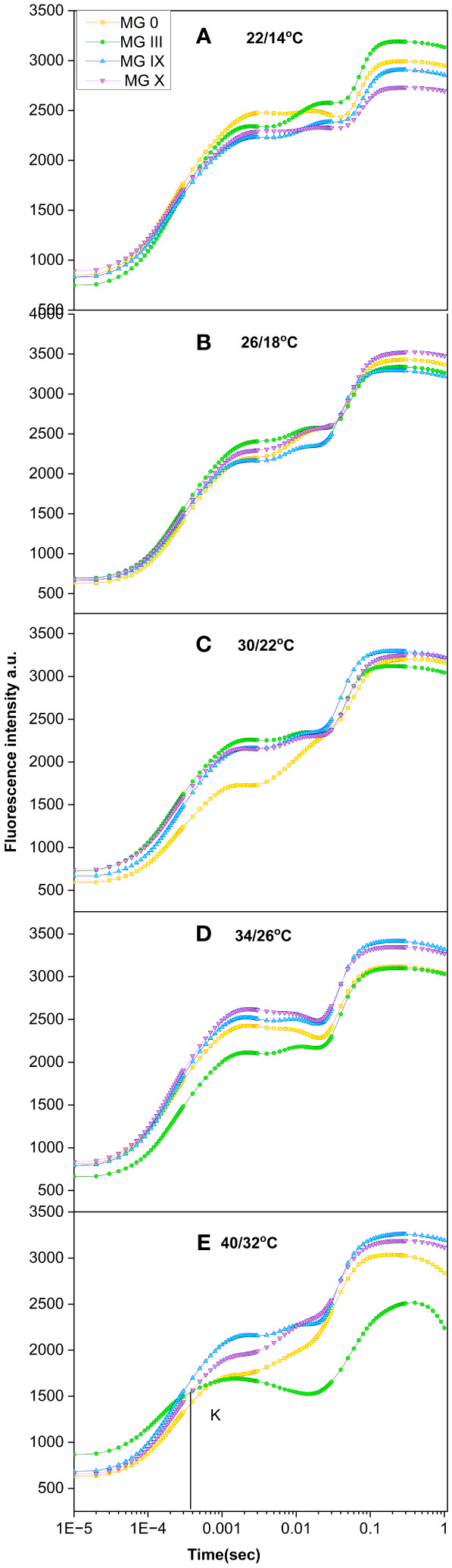
Chlorophyll *a* fluorescence transient curves for soybean early to late maturity groups at **(A)** 22/14°C, **(B)** 26/18°C, **(C)** 30/22°C, **(D)** 34/26°C, and **(E)** 40/32°C temperatures.

Soybean early and late maturity groups were studied for all the temperatures (**Figures 3A–E**). Both low and high temperatures greatly affected the transients and indicated a decline in photosynthetic activity, as observed by a decrease in some of the inflection points in OJIP (**Figures 3A, D, E**). A distinct J–P amplitude drop was obtained at low (22/14°C) and high (34/26°C and 40/32°C) temperatures. A decrease in the J–P point was designated for inhibiting electron transport through the plastoquinone pool (**Figure 3A**) at low and high temperatures. In the present study, it was not only the low day temperature but also the night temperature (22/14°C) that was partially responsible for the declining photosynthetic activity of the soybean maturity groups. A decline in the I–P phase suggested a loss of electron transport from *Q*_A_ to *Q*_B_. Previously, Mathur et al. (2021) reported a decrease in I and P amplitude in sugarcane at low temperatures (Mathur et al., 2021). A decrease in J, I point followed by a decreased Fm was observed for all the MGs at 34/26°C and 40/32°C. MG III showed a dramatic decline in Fm and an increase in Fo, respectively. At 40/32°C, a prominent additional K point was observed for MG III. At the same time, MG 0 showed a slightly diminished K point (**Figure 3E**), indicating damage to the oxygen-evolving complex (OEC) (Barboricova et al., 2022) due to high temperature. An increase in the *F*_k_/*F*_j_ ratio for 34/26 (0.74)°C and 40/32°C (0.76) indicated OEC damage, while the *F*_k_/*F*_j_ ratio for 26/18°C and 30/22°C was within the limit of 0.6. An additional H step was observed for MG III (**Figure 3E**). A fluorescence decline was observed at the P point, splitting into a steep dip as the H step. The appearance of the H point was due to the removal of limitations on the acceptor side of PS I (Ilik et al., 2006; Mathur and Jajoo, 2014). A minor H point (bump) was also observed for MG 0. This is also supported by the previous studies suggesting less photosynthesis and less photoassimilate production, followed by less photoassimilate distribution to the reproductive organs, resulting in flower or pod drop at higher temperatures (Allen et al., 2018). A prominent decline in fluorescence was observed at 34/26°C as well. The optimum temperatures of 26°C–30°C did not show any change in the transients for all the maturity groups. PSII reaction centers are connected and can distribute or redistribute energy. This is called PSII connectivity. The results at 40/32°C also displayed a disconnection in the PSII reaction centers and a loss of energy distribution. Thus, like day length curves for temperatures, the shape of Chl transients can be considered to identify any stress or climate change for soybean maturity groups.

Furthermore, we studied fluorescence parameters derived from chlorophyll *a* transient (**Table 4**). Compared to day lengths, damaging effects were prominent for most of the fluorescence parameters at high and low temperatures. *F*_v_/*F*_m_ decreased for low and higher temperatures compared to 26/18°C and 30/22°C, respectively. At low temperatures, late maturity groups showed a greater decrease in *F*_v_/*F*_m_ compared to early ones. This suggests that the conversion and capture efficiency of the electrons and the quantum efficacy decreased at low temperatures for all the maturity groups. Previously, Kruger et al. (2014) also reported a decline in soybean photosynthetic activity in response to low night temperatures. This further led to a decrease in other correlated parameters of the PSII (discussed in the later part of the manuscript). Thus, *F*_v_/*F*_m_ should be considered a potential parameter to identify stress conditions for soybean maturity groups under changing temperatures. *F*_o_/*F*_m_ (ϕ_Do_) for early and late maturity groups increased at low and high temperatures. The increase in *F*_o_/*F*_m_ indicates the plants are under stress conditions (Strasser et al., 1995). For MG 0, IX, and X, 22/14°C showed the highest *F*_o_/*F*_m_, while for MG III, a maximum increase in *F*_o_/*F*_m_ was observed at 40/32°C, indicating that plants were trying to release excessive energy in the form of heat to adjust themselves to changes in temperature regimes (**Table 4**) and to cope with stressful conditions.

**Table 4:** Chlorophyll *a* fluorescence parameter for soybean maturity groups under various temperatures.

Maturity group	Temperature (day/night) (°C)	*F*_v_/*F*_m_	*F*_o_/*F*_m_	ϕ_Eo_	δ_Ro_	PI Inst.	γ(RC)/((1-γ(RC))
0	22/14	0.734d	0.27a	0.18e	1.03a	0.33d	0.37cd
	26/18	0.843a	0.16d	0.36b	0.65b	1.60b	0.46a
	30/22	0.836a	0.16d	0.46a	0.56b	2.25a	0.42b
	34/26	0.762c	0.24b	0.22d	1.06a	0.42d	0.37d
	40/32	0.798b	0.20c	0.28c	0.99a	0.72c	0.40c
	Significance	^***^	^***^	^***^	^***^	^***^	^***^
	*p*-value	<0.001	<0.001	<0.001	<0.001	<0.001	<0.001
III	22/14	0.786a	0.21b	0.26	0.83b	0.81a	0.39b
	26/18	0.819a	0.18b	0.29	0.78b	0.94a	0.45a
	30/22	0.789a	0.21b	0.28	0.87b	0.69ab	0.39b
	34/26	0.806a	0.19b	0.29	0.89b	0.83a	0.41b
	40/32	0.739b	0.26a	0.23	1.09a	0.42b	0.34c
	Significance	^**^	^**^	ns	^*^	^*^	^***^
	*p*-value	0.002	0.002	0.283	0.013	0.018	<0.001
IX	22/14	0.733c	0.27a	0.24b	0.78b	0.49b	0.36c
	26/18	0.823a	0.18c	0.39a	0.75b	1.60a	0.46a
	30/22	0.824a	0.18c	0.34a	0.70b	1.23a	0.43b
	34/26	0.794b	0.21b	0.26b	0.89a	0.60b	0.38c
	40/32	0.790b	0.21b	0.28b	0.76b	0.74b	0.36c
	Significance	^***^	^***^	^***^	^***^	^***^	^***^
	*p*-value	<0.001	<0.001	<0.001	<0.001	<0.001	<0.001
X	22/14	0.691c	0.31a	0.17c	0.89b	0.24c	0.34b
	26/18	0.830a	0.17c	0.35a	0.73c	1.44a	0.46a
	30/22	0.753b	0.25b	0.27b	0.93b	0.56b	0.36bc
	34/26	0.783b	0.22b	0.24b	0.92b	0.52b	0.38b
	40/32	0.761b	0.24b	0.18c	1.11a	0.32bc	0.35bc
	Significance	^***^	^***^	^***^	^***^	^***^	^***^
	*p*-value	<0.001	<0.001	<0.001	<0.001	<0.001	<0.001

Significance was determined at ^***^p ≤ 0.001; **p ≤ 0.01; ^*^p ≤ 0.05. Mean with the same letter are not significantly different.

Linear electron transport and the electron movement after *Q*_A_− represented by φ_Eo_ decreased for low and high temperatures, suggesting that high temperatures directly impact the donor side while low temperatures affect the acceptor side of PSII, respectively (**Table 4**). Individually, electron transfer or movement was hindered for all the maturity groups, but maturity group MG X reflected maximum decline at low and high temperatures, respectively. An increase in δ_Ro_ at high and low temperatures indicated that electrons could not be transferred efficiently to PSI. Thus, it can be said that temperatures impacted the donor and acceptor sides of PSII. The performance of all maturity groups at various temperatures can be studied using the parameter PI_ins_. (Performance index instrument or total). A significant decline in PI_ins._ at 22/14°C and 40/32°C, indicating reduced absorption, trapping, and lesser electron availability at the PSII donor and acceptor sides, followed by final electron transfer to PSI. A decline of PI at low temperatures was also correlated with chlorophyll and carotenoid breakdown (Ferrante and Maggiore, 2007; Stirbet et al., 2018). The measure of reaction center density of active reaction centers with the chlorophyll bed γ(RC), represented as [γ(RC)/((1-γ(RC))], also decreased for low and high temperatures for all the maturity groups (**Table 4**), indicating that lesser active chlorophyll reaction centers were available at the low and high temperatures. Due to low and high temperatures, the active RCs were converted to less efficient or nonefficient RCs. Thus, changes/alterations in reaction centers were also one temperature parameter to consider.

### Impact of temperature on derived parameters, flux ratios, quantum yields, and efficiencies

**Figure 4:**
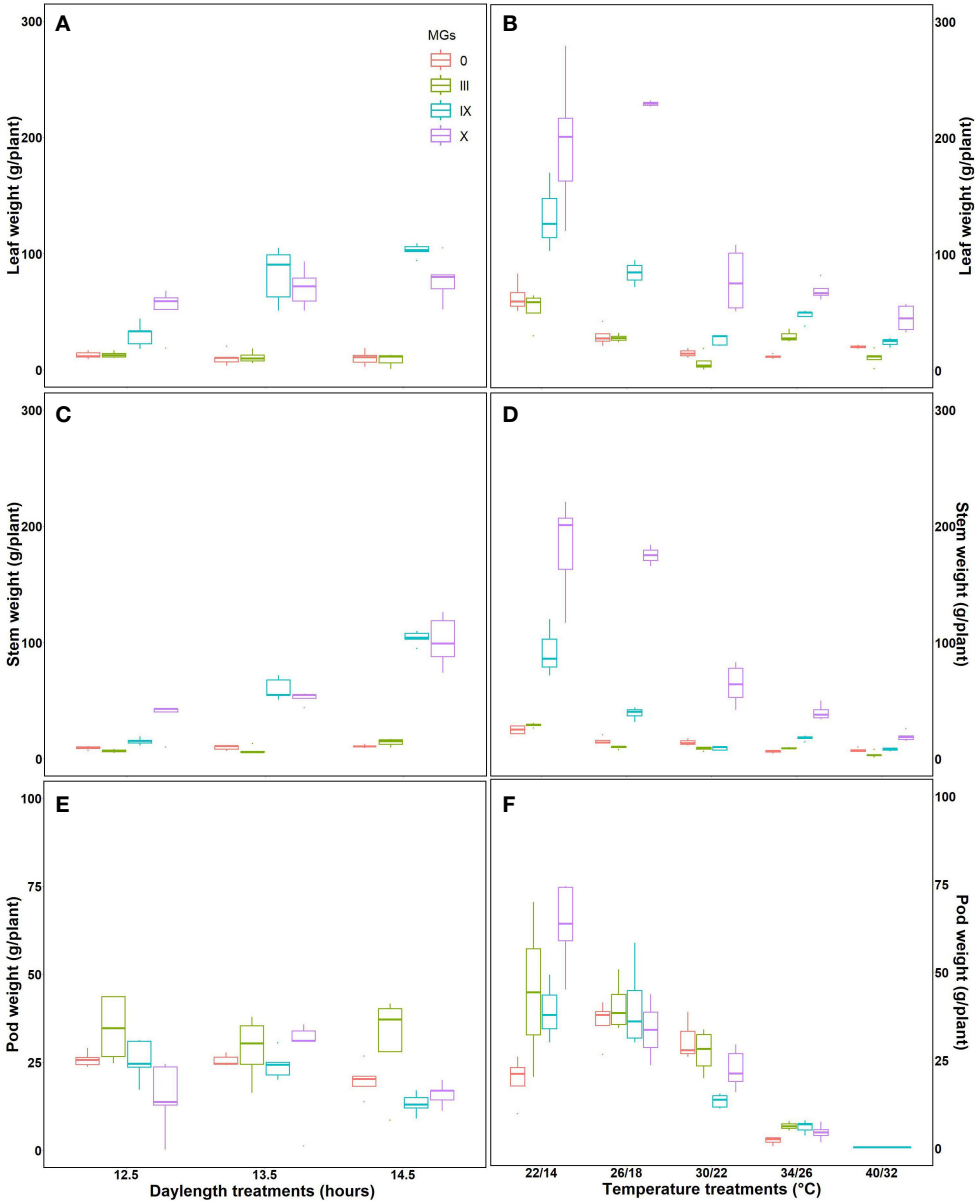
Measured flux ratios **(A**, **B)**, yield parameters **(C**, **D)**, deexcitation nonphotochemical and photochemical rate constants **(E**, **F),** and vitality index and forces **(G**, **H)** for early to late soybean maturity groups under changing temperatures, respectively.

Derived parameters, flux or yield ratios, specific fluxes, quantum yield and efficiencies, and deexcitation rate constants (**Table 2**) were studied for different temperatures and maturity groups (**Figures 4A–H**). For yield efficiencies, as compared to 26/18°C and 30/22°C, an increase in the TRo/RC ratio was observed at 22/14°C, 34/26°C, and 40/32°C, respectively (**Figure 4A**). The increase depicted the high efficiency of reaction centers to reduce plastoquinone. Trapping increased, but not all trapped excitons were efficiently converted and transported to the electron transport chain (Chen and Cheng, 2009; Snider et al., 2018). REo/RC decreased for low and high temperatures compared to optimum temperatures. MG 0 showed a maximum decline for REo/RC, which suggests that the damage due to low and high temperatures was limited (not much) at the PSI end electron acceptor. Temperatures caused PSII and linear electron transport more damage than PSI (**Figure 4B**). Increased TRo/RC and REo/RC values also indicate a partial decrease in the number and activity of active PSII reaction centers. Thus, REo/RC can be a stress indicator for the maturity groups.

Ψ_Eo_ and φ_Ro_ decreased for 22/14°C, 34/26°C, and 40/32°C temperatures. This decrease in Ψ_Eo_ suggests that the chance of an exciton that can move an electron further than *Q*_A_ was limited due to low and high temperatures (**Figures 4C, D**). Similarly, φ_Ro_ values also reduced, implying that the quantum yield of electron transport from *Q*_A_− to PSI end electron acceptor decreased because of day and night temperature impact. The parameters φ_Ro_, REo/RC, δ_Ro_, etc. provide information for the photosynthetic apparatus’s PSI side. It can be said that compared to PSII, the damage to PSI was less. MG 0, III, and X were more negatively affected. During the breeding program or selecting the tolerant and susceptible maturity groups for low and high temperatures, the parameters Ψ_Eo_ and REo/RC can be chosen for MG 0 and X. All the studied MGs are more responsive to Ψ_Eo_.

Deexcitation rate constants of the PSII antenna were studied by measuring *K*_P_ and *K*_N_ for all the temperatures and maturity groups. It was observed that the value of *K*_P_ decreased while that of *K*_N_ increased for temperatures (low and high) (**Figures 4E, F**). This increase in the deexcitation rate constant for photochemical reactions indicated that the plant tried confiscating additional heat from the system, thus decreasing the deexcitation nonphotochemical rate constant (*K*_N_) (**Figures 4E, F**). Out of *K*_P_ and *K*_N_, *K*_P_ can be considered a parameter for different maturity groups.

We also studied the photosynthetic driving force (DF_total_) (Srivastava et al., 1999; Mathur and Jajoo, 2015) structural–functional index (SF_abs_) of plants (**Figures 4G, H**) (Dudeja and Chaudhary, 2005; Mathur and Jajoo, 2015). All these parameters represent the soybean maturity groups’ health status at various temperatures. It was observed that DF_total_ was more damaging and sensitive to higher as compared to lower temperatures for all the MGs. MG 0, III, and X were highly impacted maturity groups; 26/18°C and 30/22°C presented maximum values for both indices. Reduced DF_total_ indicated that low and high temperatures caused a decrease in the driving force of photosynthesis (such as absorption, trapping, and transport of electrons across or beyond *Q*_A_−). DF_total_ can be considered one of the determining parameters for the overall health of photosynthetic apparatus as it provides information on all the components of the light reaction responsible for channeling the dark reaction and further yield and development of the crop. Early and late maturity groups are sensitive to this parameter; therefore, this parameter can be used for a broad range of maturity groups. Different MG showed different responses to SF_abs_. For MG 0 and X, the highest decrease at SF_abs_ was observed at 22/14°C followed by 40/32°C, respectively. MG III showed a maximum decline at 40°C for SF_abs_. MG IX showed almost equal damage at lower and higher temperatures for SF_abs_, respectively (**Figures 4G, H**). This indicated that absorption was decreased for the temperatures causing damage at the thermal reactions and influencing the dark responses of PSII as well. All these results showed that for some maturity groups, the quantum efficacy of intersystem electron transport is more susceptible to low temperature than the quantum efficacy of electron transport at the PSI end acceptor. Lower night temperature is also a factor in declined quantum yields and the effectiveness of PSII and PSI.

### Effect on specific leaf area, the aboveground dry matter under day length and temperatures

**Figure 5:**
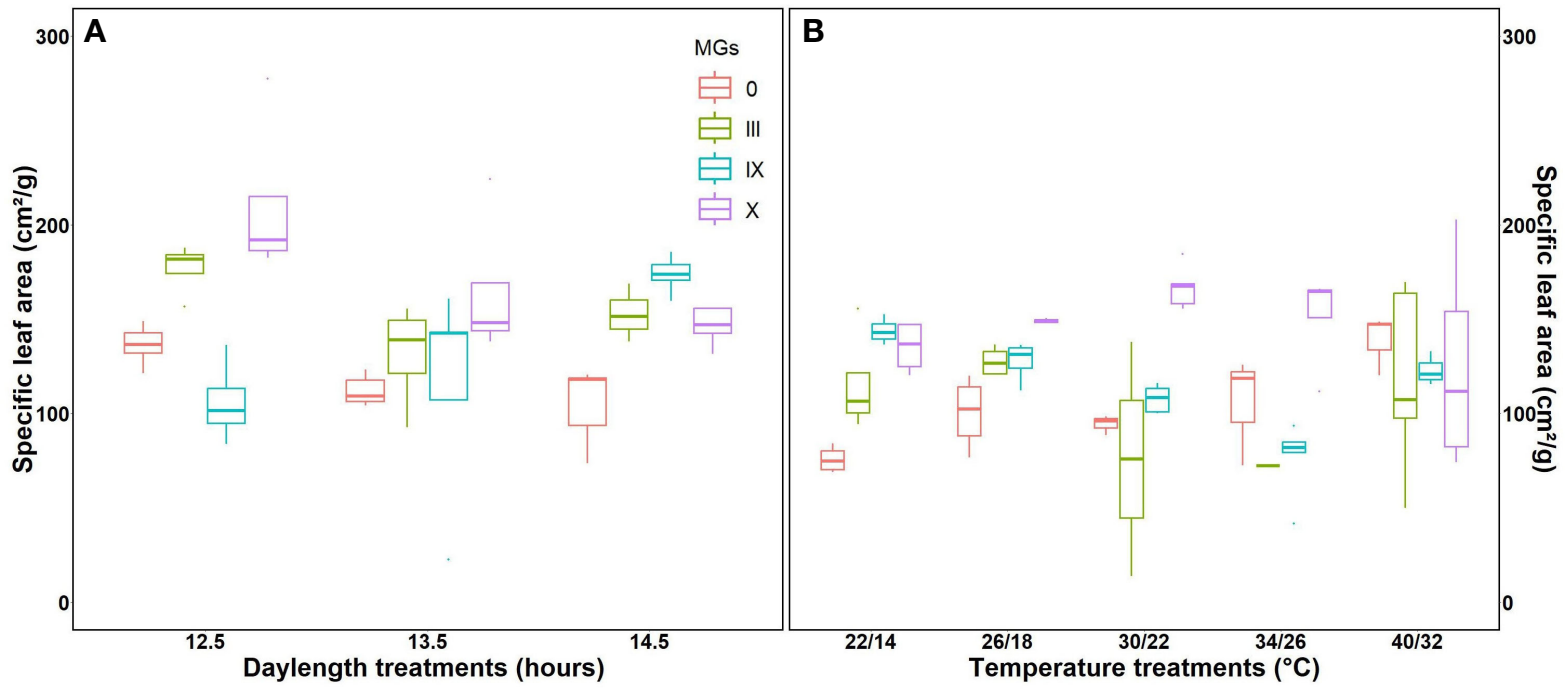
Specific leaf area for soybean early to late maturity groups at changing day lengths **(A)** and temperatures **(B)**.

Specific leaf area is directly related to the morphological characteristics of the leaf, such as thickness. The highest specific leaf area was observed for MG X at a 12.5-h day length (**Figure 5A**), followed by MG III and IX, respectively. Higher specific leaf area corresponds to increased photosynthesis, resulting in improved aboveground biomass. This was evident from our results. Evidence from fluorescence induction transients also supports that photosynthesis was better at 12.5-h day lengths. At 14.5-h DLs, SLA decreased (**Figure 5A**). We further studied specific leaf areas for various temperatures. MG 0 showed minimum SLA at 22/14°C, while MG III and IX showed the lowest values at 34/26°C, respectively (**Figure 5B**). The apparent leaf area increased at high temperatures because 40/32°C stress made the leaf more spongy, thick, and mature (strategies of plants to escape stress).

**Figure 6:**
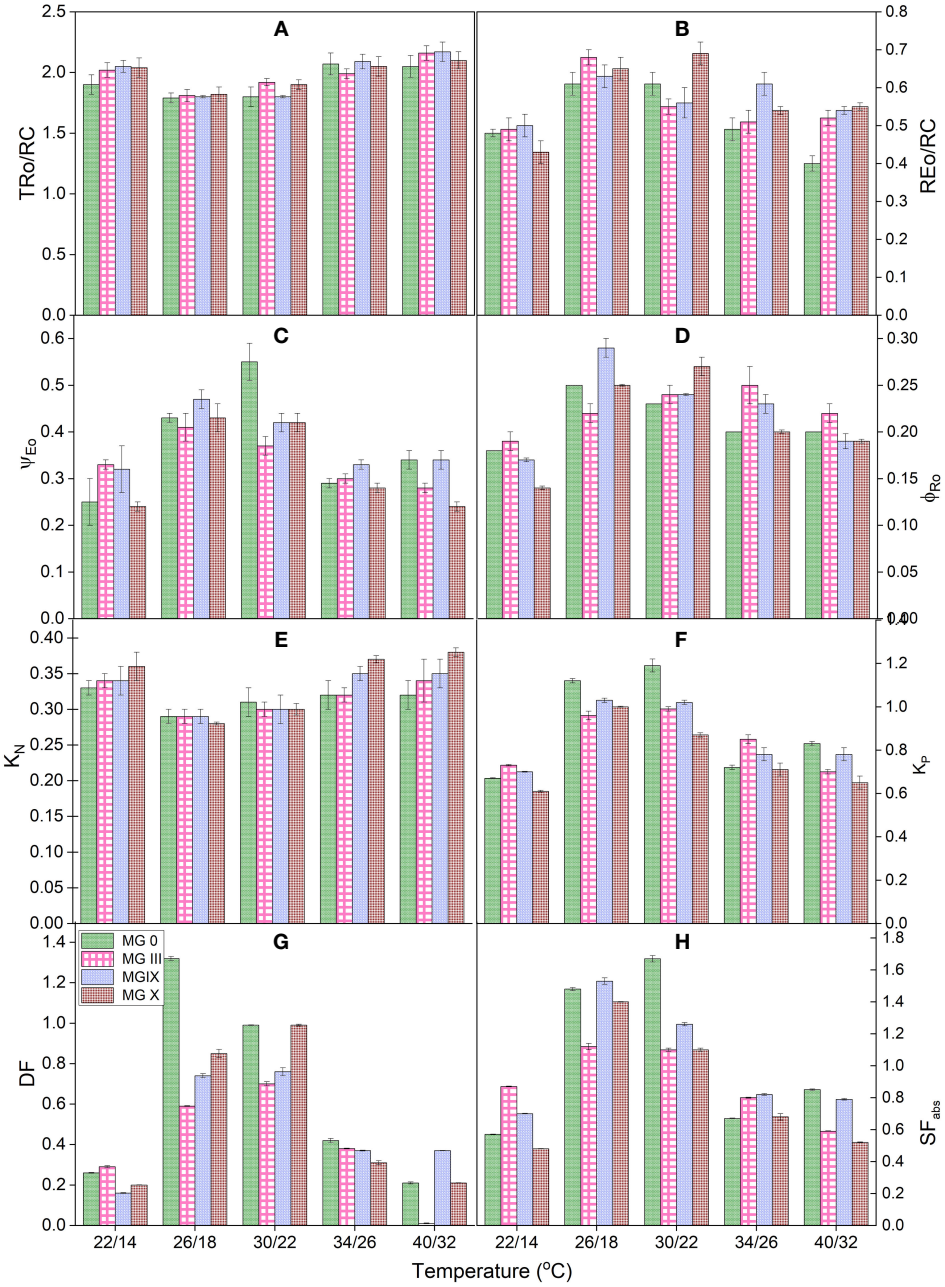
Measured aboveground dry mass for soybean early to later maturity groups under changing day lengths and temperatures **(A, B)** leaf dry weight, **(C, D)** stem dry weight, and **(E, F)** pod dry weight for soybean maturity groups under various day lengths **(A–C)** and temperatures **(D–F)**.

Aboveground dry weights were studied at harvest time for leaves, stems, and pods for various day lengths and temperatures (**Figures 6A, B**). MG 0 and III showed no significant difference in the leaf dry weight for all the day lengths. A significant difference in leaf dry weight was observed for MG IX and X at long day lengths. Stem dry weight increased with increasing day lengths for the late maturity groups, evident from increased leaf dry weight at longer day lengths. The highest pod weight was observed for early maturity groups. Individually, MG III had maximum pod weight at a 12.5-h day length followed by long day lengths (**Figure 6A**).

The leaf dry weight decreased with increasing temperatures, which is in support of the fluorescence studies, which showed decreased primary photochemistry. The highest stem dry weight for MG X was observed for 22/14°C. The stem dry weight decreased at 34/26°C and 40/32°C, respectively. MG 0, IX, and X showed maximum pod weight at 26/19°C, followed by 30/22°C. The pod weight decreased as the temperature increased. No or aborted pods were obtained at 40/32°C, respectively (**Figure 6B**). The aboveground dry weight measurements supported the fluorescence measurements indicating the highest photosynthesis for plants grown at optimum temperature conditions.

### Correlation and heat map of the studied fluorescence parameters

**Figure 7:**
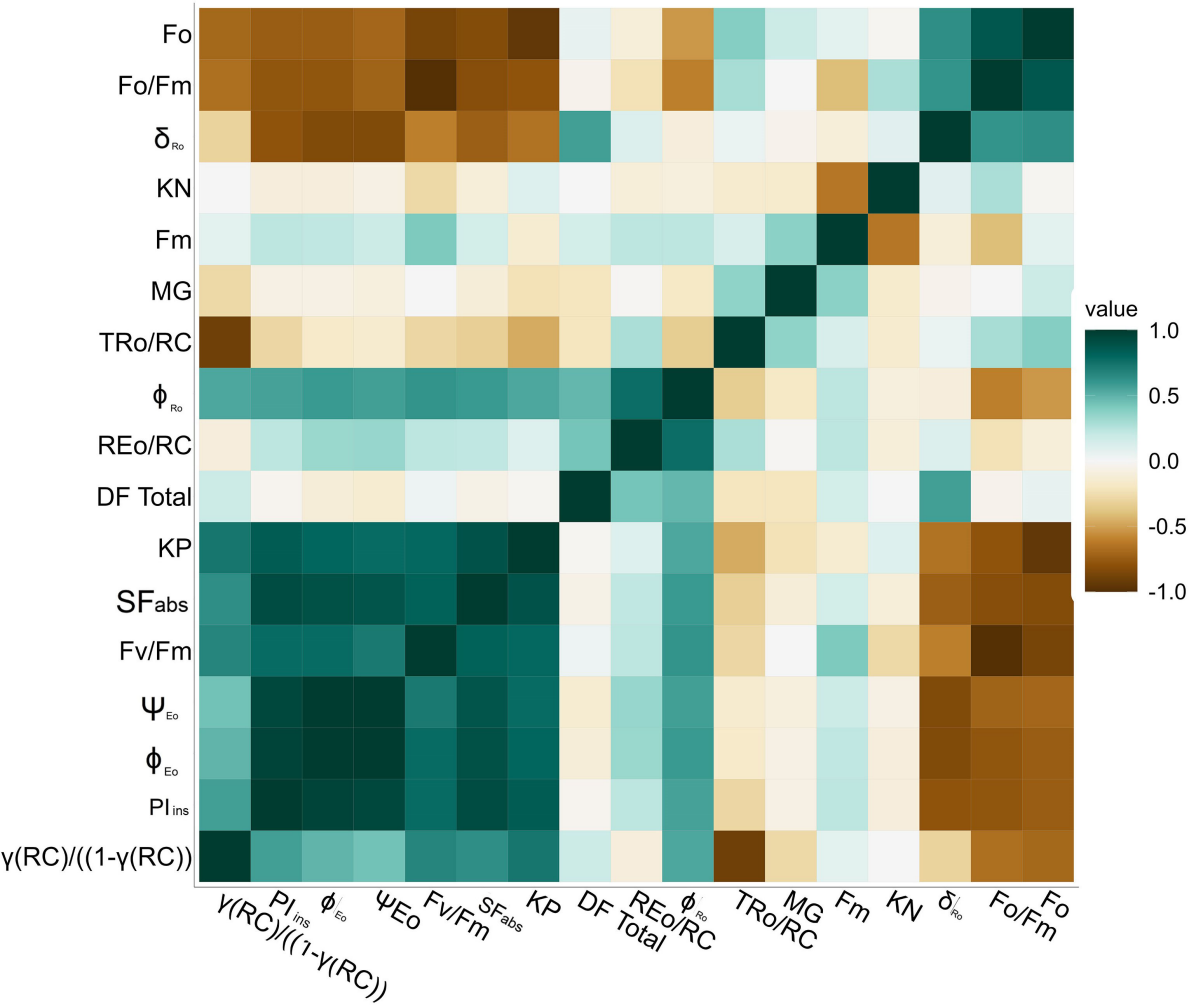
Correlation matrix (heat map) for chlorophyll *a* fluorescence parameter with color code for soybean maturity groups, different day lengths, and temperature.

A heatmap generated a correlation to study the relationship between fluorescence parameters for early to late soybean maturity groups for day lengths and temperatures (**Figure 7**). The heatmap used a color scheme to represent the magnitude and direction of correlations between parameters. The color’s intensity or saturation reflects the correlation’s strength, with higher saturation indicating stronger correlations and lower saturation indicating weaker correlations. Dark teal (dark green) and brown indicate positive and negative correlations, respectively. The dark teal color showed a positive, whereas the brown indicated a negative correlation. As the color becomes progressively brighter, it represents a decrease in correlation (**Figure 7**). According to the graph, the coefficients were divided into three groups related to *F*_o_, *F*_m_, and *F*_v_/*F*_m_, respectively. High correlations were observed among several coefficients, including *F*_v_/*F*_m_ and Ψ_Eo_, while the *F*_o_, *F*_o_/*F*_m_ parameters displayed high correlation coefficients.

Additionally, strong correlations were identified between *F*_m_, while the *F*_o_, *F*_o_/*F*_m_, coefficients displayed high correlation coefficients. However, the *F*_v_/*F*_m_ group and the *F*_o_ group exhibit a negative correlation with each other. Additionally, parameters such as KN and TRo/RC showed weak correlations with the other parameters. This correlation will be helpful for future studies. Based on positive or negative interactions, we can decide the traits required to identify soybean-tolerant or sensitive in-breed lines. These parameters or traits will also help to determine the status of light reactions for PSII for soybean plants and improve Farquhar equations for the soybean photosynthesis model.

## Conclusion

The current study provides information about alterations in the light reactions of early and late soybean maturity groups under varied day lengths and temperatures. This study is an effort to fill the knowledge gap existing about Chl *a* fluorescence usage for light reactions in response to stress at early or any stage of plant life. The shape of the chlorophyll curve can be considered one of the major deciding traits for studying any day length and maturity group. Day lengths might not impact the plant, but they affect the photosynthetic activity observed in our present study. Each maturity group behaved differently for a particular day length. Based on this study, MG X proved very sensitive for long day lengths and can be further used as a reference maturity group. In terms of temperature response, soybean maturity groups 0, III, and X displayed sensitivity to varying temperature conditions, corresponding to a distinct chlorophyll curve shape. The K point indicated damage to the oxygen-evolving complex of PSII. Low temperature caused damage at the acceptor side of PSII and decreased the electron movement further from PSI to the end electron acceptor. High temperatures and low temperatures were equally damaging to crop yield and development. The SLA and above-ground dry mass measurement data also supported the fluorescence study. Identifying the traits that can be used for temperatures is easy but tricky for day lengths. Therefore, it is concluded that chlorophyll *a* fluorescence could be used as a potential indicator or technique for studying temperature and day-length responses in the soybean’s early- and late-maturity groups. Moreover, the method is faster and, therefore, can be used widely in agricultural fields. It can be encouraged as a major high-throughput phenotyping technique that will be nondestructive. The study will also help to improve the existing soybean simulation models. The study can add more information to the current Farquhar photosynthesis model since the fluorescence measurements provide details about the light reactions. This study will benefit breeders in selecting tolerant and susceptible genotypes to identify any abiotic stress at a very early stage of plant life to avoid damage in yield.

## Data availability statement

The original contributions presented in the study are included in the article/**Supplementary Material**. Further inquiries can be directed to the corresponding author.

## Author contributions

VR and SM conceived the study. SM and VR designed the experiments. SM and BS conducted the experiment and collected the data. SM and BS analyzed the data. SM wrote the initial draft. VR, AJ, and KR reviewed and edited the manuscript. All authors contributed to the article and approved the submitted version.

## Supplementary material

Supplementary Figure 1 The light response curve of soybean plants at various light intensities at 40/32°C.
