# Supplementary figures and images for: Chlorophyll fluorescence is a potential indicator to measure photochemical efficiency in early to late soybean maturity groups under changing day lengths and temperatures
Source: Front Plant Sci. 2023 Oct 23;14:1228464. doi: 10.3389/fpls.2023.1228464 (PMC10627226; doi:10.3389/fpls.2023.1228464)

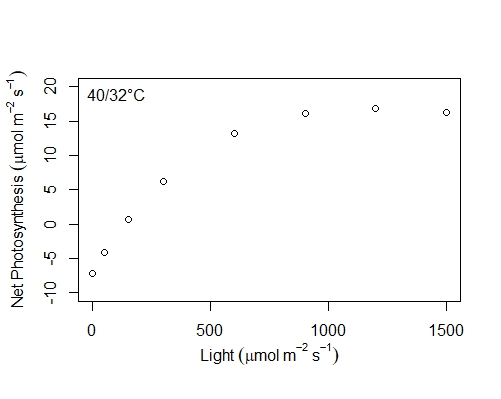

Supplement: Supplementary Figure 1 — The light response curve of soybean plants at various light intensities at 40/32°C. [file Image_1.jpeg]
